# Epigenetic inactivation of *VGF* associated with Urothelial Cell Carcinoma and its potential as a non-invasive biomarker using urine

**DOI:** 10.18632/oncotarget.1949

**Published:** 2014-05-07

**Authors:** Masamichi Hayashi, Heike Bernert, Luciane Tsukamoto Kagohara, Leonel Maldonado, Mariana Brait, Mark Schoenberg, Trinity Bivalacqua, George J Netto, Wayne Koch, David Sidransky, Mohammad O. Hoque

**Affiliations:** ^1^ Department of Otolaryngology-Head and Neck Surgery, The Johns Hopkins University School of Medicine, Baltimore, Maryland, USA; ^2^ Department of Pathobiology, The University of Tennessee, The College of Veterinary Medicine, Knoxville, Tennessee, USA; ^3^ Department of Gynecology and Obstetrics-Gynecologic Specialties, The Johns Hopkins University School of Medicine, Baltimore, Maryland, USA; ^4^ Department of Urology, The Johns Hopkins University School of Medicine, Baltimore, Maryland, USA; ^5^ Department of Pathology, The Johns Hopkins University School of Medicine, Baltimore, Maryland, USA; ^6^ Department of Oncology, The Johns Hopkins University School of Medicine, Baltimore, Maryland, USA

**Keywords:** UCC, Epigenetics, Biomarker, Methylation

## Abstract

Background: To identify new epigenetic markers and further characterize Urothelial Cell Carcinoma (UCC), we tested the promoter methylation (PM) status of 19 genes previously identified as cancer specific methylated genes in other solid tumors.

Methods: We used bisulfite sequencing, methylation specific PCR and quantitative methylation specific PCR (QMSP) to test the PM status of 19 genes in urothelial cancer cell lines.

Results: Among the 19 genes tested, *VGF* was found to be completely methylated in several UCC cell lines. *VGF* QMSP analysis showed that methylation values of almost all the primary 19 UCC tissues were higher than the paired normal tissues (P=0.009). In another cohort, 12/35 (34.3%) of low grade UCC cases displayed *VGF* methylation. As a biomarker for non-invasive detection of UCC, *VGF* showed a significantly higher frequency of methylation in urine from UCC cases (8/20) compared to controls (1/20) (P=0.020). After treatment of cell lines with 5-Aza-2'-deoxycytidine, VGF was robustly re-expressed. Forced expression of VGF in bladder cancer cell lines inhibited cell growth.

Conclusion: Selection of candidates from genome-wide screening approach in other solid tumors successfully identified UCC specific methylated genes.

## INTRODUCTION

Globally, 2.7 million people are assessed to be diagnosed with, or have a history of urothelial cell carcinoma (UCC) [1]. In the USA, more than 72,000 new cases of, and over 15,000 deaths from UCC of the urinary bladder are estimated in 2014 [2]. UCC is an extremely heterogeneous disease and there are notable stage-dependent differences in survival rates for patients with localized or metastatic tumors. Current data released from the National Cancer Institute (NCI) shows that the relative 5-year survival rate for patients with stage I-IV disease is 96.6%, 70.7%, 34.6%, and 5.4%, respectively[3]. Of all UCC, the majority (75–85%) presents as non-muscle-invasive bladder cancer (NMIBC) confined to the mucosa [stage Ta in 70%; carcinoma *in situ* (CIS) in 10%] or to the sub-mucosa (stage T1 in 20%) [3-5]. These NMIBC lesions recur quite frequently (70-80%) presenting a significant problem in the overall management and monitoring of the disease [5]. NMIBC tumors range from benign low-risk, low grade tumors to high-risk, high-grade tumors that are almost certain to recur and have a significant risk of progression to muscle-invasive or metastatic disease [4]. Therefore, effective UCC treatment requires accurate detection and long-term monitoring. Clinicopathological prognostic factors that are currently used to predict the course of this disease are of moderate utility [6]. In order to manage UCC in a cost effective manner, it is required to understand the biology of this disease and to establish tumor specific biomarkers for early cancer detection and monitoring.

Recently, we identified *VGF* as a cancer specific methylated gene in our “Cancer Methylome” discovery approach [7] and also reported *VGF* as an ovarian and testicular cancer specific methylated gene by candidate gene approach [8] [9], a strategy which was used in the present study. *VGF* gene is located at chromosome 7 q22.1 and has been shown to play an essential role in body weight, basal metabolism, nutrition [10], regulating energy homeostasis [11] and strengthening synaptic system associated with learning and memory [12]. Beyond the report of inactivation of *VGF* in cancer by our group, it was also reported by other groups as a gene associated with different pathologic conditions; such as mutation of *VGF* in mice produced depression [13] and homozygous VGF-null mice were small, hypermetabolic, hyperactive, and infertile [11]. Deregulation of *VGF* associated gene was reported in bladder tissue of interstitial cystitis [14, 15]; however, direct correlation of *VGF* with UCC is still not reported.

For about two decades, methylation changes, both globally and gene specifically, have been known to be associated with different pathological conditions, particularly cancer. There are numerous microarray-based & high throughput sequencing techniques integrated with sophisticated computational approaches are available to measure cytosine methylation across the genome, as well as gold-standard techniques based on sequencing bisulfite converted DNA, which is used to measure methylation in a smaller and more targeted set of loci. All of these techniques and approaches have advantages and limitations to identify accurate and specific methylation of a locus. We recently reported a robust approach that couples genome-wide probabilistic search algorithms with an established pharmacologic unmasking strategy for unbiased and precise global localization of tumor-specific methylated genes that include 5 major cancer types excluding UCC [7]. By this comprehensive approach, a set of 175 novel candidate genes was identified, which clustered throughout the genome and harbored cancer-specific promoter methylation. A significant number of these genes have already been tested in different types of cancer by our group and others [8, 9, 16-19]. Among the 175 genes that were discovered as a cancer-specific methylated genes by our integrated approach, we only tested eight genes in UCC in our previous study [7]. To understand the expanded spectrum of “UCC Methylome”, here we analyzed 19 genes in urothelial cancer by candidate gene approach to evaluate new promising UCC specific methylated genes. Among the UCC specific methylated genes identified, *VGF* was found to have a high frequency of methylation in primary UCC, which was determined by quantitative methylation specific PCR (QMSP). Strong anti-proliferative activity of *VGF* was also observed in bladder cancer cell lines. Furthermore, we identified high frequency of methylation of *VGF* in primary low-grade papillary urothelial cell carcinoma (LGUCC). The result supports that *VGF* promoter methylation may be an early event in this disease initiation. More interestingly, *VGF* methylation can be detected in urine with high sensitivity and specificity.

## RESULTS

In order to minimize complexity of analysis in our previous integrated approach [7], we used 5 major cancer types (Lung, colon, cervix, prostate and breast) and were not able to analyze the methylation patterns in other tumors including UCC. Here we tested 19 cancer specific methylated genes based on the criteria noted in the material and method section.

### Methylation frequency of 19 candidate genes in Bladder cancer cell lines

The methylation frequency and patterns of each of the 19 genes were evaluated by bisulfite sequencing in 6 to 7 bladder cancer cell lines. Methylation of a given gene was defined as “methylation positive” when at least ≥ 50% of total CpG sites within the amplified region of the promoter are methylated. Based on the later criteria, methylation positive bladder cancer cell lines were ranged from 16.7% to 100%. Methylation frequency of all analyzed genes in bladder cancer cell lines, gene symbol, gene name and chromosomal localization are summarized in Supplemental Table S4. Representative chromatograms of bisulfite sequencing of selected genes are shown in Supplemental Figure S1.

### Association of promoter methylation with expression of selected genes in bladder cancer cell lines; and primary tumor with paired normal urothelial tissues

**Figure 1:**
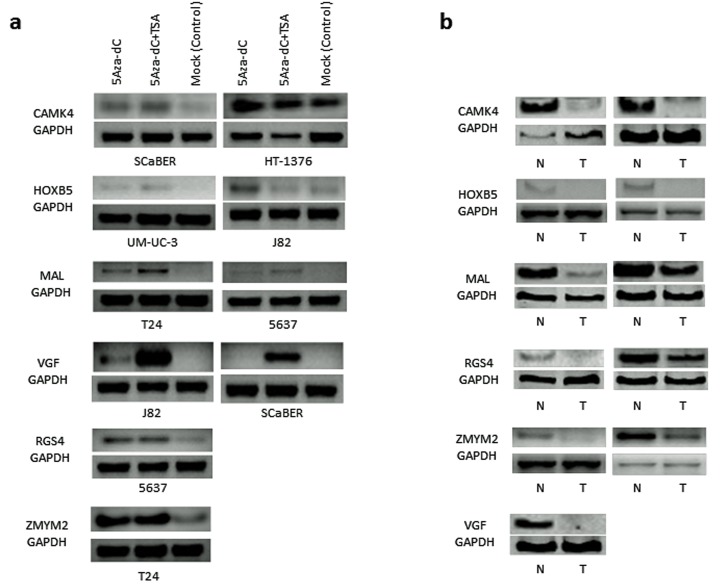
Pharmacological unmasking analysis of 6 selected genes (*CAMK4, HOXB5, MAL, RGS4, VGF* and *ZMYM2*) that showed high frequency of methylation in bladder cancer cell lines **a.** Bladder cancer cell lines were selected if the specific gene's promoter region was methylated by bisulfite sequencing and/or conventional MSP. Re-expression of each gene was observed by pharmacologic unmasking using 5μM 5-Aza-dC treatment alone or in combination with 300 nmol Trichostatin A (TSA) for 5 days. Control sample including the same volume of DMSO in PBS. Re-activation of each gene at least in one cell line was observed after 5-Aza-dC treatment. Robust expressions of some genes (e.g. *VGF*) were observed after the combination treatment of 5-Aza-dC and TSA. **b.** Expression of all the 6 genes was markedly down-regulated in primary tumor tissue (T) as compared to corresponding normal tissue (N). *GAPDH* expression is shown as an internal control at the bottom. N: Normal bladder sample, T: bladder cancer tissue.

Based on the methylation frequency and patterns; and known functional characteristics, we selected 6 genes (Table 1) for pharmacologic unmasking study using bladder cancer cell lines. To this end, different bladder cancer cell lines with promoter methylation of each of the gene were treated with 5-Aza-dC with or without Trichostatin A (TSA). Each of the 6 gene's expression was robustly reactivated by the demethylation agent 5-Aza-dC at least in one of the bladder cancer cell line (Figure 1a, Supplemental Table S5). The reactivation was stronger for some genes when both 5-Aza-dC and 300 nmol/L TSA were used in combination. To determine the expression of all the 6 genes in primary tumor tissues with paired normal, we performed semi-quantitative RT-PCR in a limited numbers of samples. As expected, expression of each of the 6 genes was silenced or reduced in primary UCC compared with corresponding normal tissues (Figure 1b). These results indicated that all of the analyzed candidate genes were at least partially methylated and silenced in UCC.

**Table 1:** Known or proposed function of selected candidate genes with high frequency of methylation in bladder cancer cell lines based on bisulfite sequencing

RefSeq	Gene Symbol	Known or Proposed Function
NM_001744	CAMK4	multifunctional serine/threonine protein kinase; transcription activator activity; nucleotide binding; transferase activity
NM_002147	HOXB5	sequence-specific DNA binding; transcription factor activity
NM_002371	MAL	apoptotic protease activator activity; channel activity; lipid binding
NM_005613	RGS4	GTPase activator activity; calmodulin binding; negative regulation of signal transduction; inactivation of MAPK activity
NM_003378	VGF	growth factor activity; neuropeptide hormone activity
NM_003453	ZMYM2	activation of AKT and MAPK pro-survival signaling pathways; regulation of transcription; metal ion binding; protein binding; zinc ion binding

### QMSP of VGF in urothelial carcinoma and urine samples

**Figure 2:**
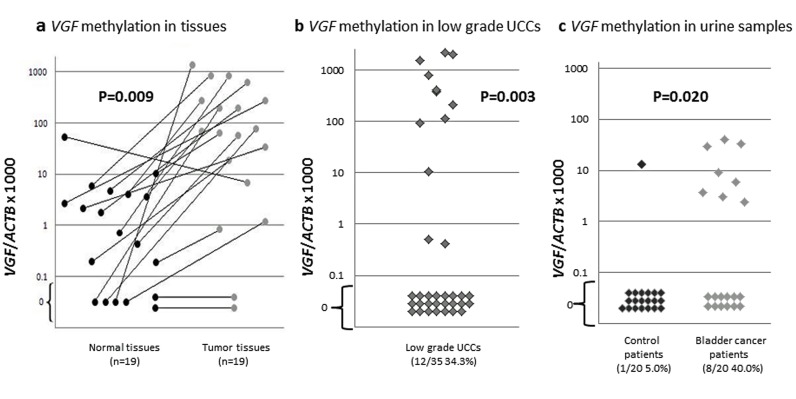
Quantitative methylation specific PCR (QMSP) of *VGF* in primary UCC and normal urothelium **a.** QMSP of *VGF* on 19 primary UCC and matched normal urothelial tissue samples. The relative level of methylated DNA for *VGF* in each sample was determined as a ratio of QMSP for the *VGF* gene to *β-actin*, multiplied by 1000 for easier tabulation. Statistical analysis was performed using Student's t-test to determine the differences between tumor-normal matched samples; b: Relative QMSP values of *VGF* in 35 low grade UCCs were displayed. Over 34% of low grade UCCs had *VGF* methylation. Values designated below 0.1 are zero values, which cannot be plotted correctly on a log scale. Statistical analysis was performed using Fisher Exact test to determine the differences between normal tissues (n=19 in Figure 2a) and LGUCC cases. The cut off value was determined as a maximum QMSP value of normal tissues (Figure 2a, left panel) in order to emphasize on the cancer specificity. *VGF* promoter methylation was statistically significant in LGUCC cases (p=0.003); c. Quantitative methylation-specific polymerase chain reaction (PCR) methylation levels of *VGF* in urine sediment DNA of UCC patients (UCC cases, n=20) and subjects with no known neoplastic disease (controls; n=20). Calculation of the *VGF* promoter methylation/*β-actin* ratios was based on the fluorescence emission intensity values for both the *VGF* and *β-actin* obtained by quantitative real-time PCR analysis. The relative frequency of methylated promoter DNA was much higher in urine sediment from UCC patients compared with controls. Values designated below 0.1 are zero values, which cannot be plotted correctly on a log scale.

Taken the advantage of optimized QMSP primers and probes from our previous studies [8, 9] and our initial observation of UCC specific methylation and silencing of *VGF*, we decided to analyze an extended number of clinical samples for *VGF* gene. To this end, we tested a total of 19 tumors with matched normal tissues. As shown in Figure 2a, relative QMSP values of tumors were significantly higher than those of matched normal tissues (P=0.009, Student's t-test). Out of 19 primary UCC and matched normal samples, 16 showed higher methylation values in tumor than normal tissues. To determine whether *VGF* methylation is an early event in urothelial carcinogenesis, we tested *VGF* in a cohort of 35 low grade papillary UCCs (LGUCC). In this cohort as well, promoter methylation of *VGF* was observed at a reasonably high frequency [12 of 35 LGUCC cases (34.^3^%)] (Figure 2b). The promoter methylations of *VGF* in LGUCC cohort were significantly higher than those of 19 normal (non-neoplastic) subjects that are shown in Figure 2a. Statistical analysis was performed using Fisher Exact test and an empiric cut off value of QMSP to determine the differences between LGUCC cases with 19 non-neoplastic cases. The cut off value was determined as a maximum QMSP value of normal tissues in order to put emphasis on the specificity of cancer detection. By this cut off value, *VGF* promoter methylation was determined in 9 out of 35 of LGUCCs and in 0 out of 19 non-matched, non-neoplastic samples (p=0.003, Fisher Exact test) (Figure 2a and 2b). These finding indicates that *VGF* promoter methylation could be an early event in a subset of LGUCCs. We further explore the feasibility of *VGF* promoter methylation detection in urine for non-invasive detection of UCC for disease monitoring and early detection. Clinicopathological data of urine samples were summarized in Supplemental Table S6. While promoter methylation of *VGF* was detected in 8/20 (40.0%) urine samples of the UCC patients, only 1/20 (5.0%) urine samples of healthy controls showed *VGF* promoter methylation (p=0.020, Fisher's exact test) (Figure 2c, Table 2). So, sensitivity and specificity of *VGF* promoter methylation of non-invasive detection of UCC is 40.0% and 95.0% respectively.

**Table 2:** Summary of VGF gene QMSP data of urine samples

Urine samples	VGF gene QMSP positive	VGF gene QMSP negative	P value	
UCC patient (n=20)	8	12	0.020 (Fisher's exact test)	Sensitivity 8/20 (40.0%)
Healthy control (n=20)	1	19		Specificity 19/20 (95.0%)

UCC: Urothelial Cell Carcinoma

### Inhibition of bladder cancer cell growth *in vitro* and colony formation by VGF over-expression

**Figure 3:**
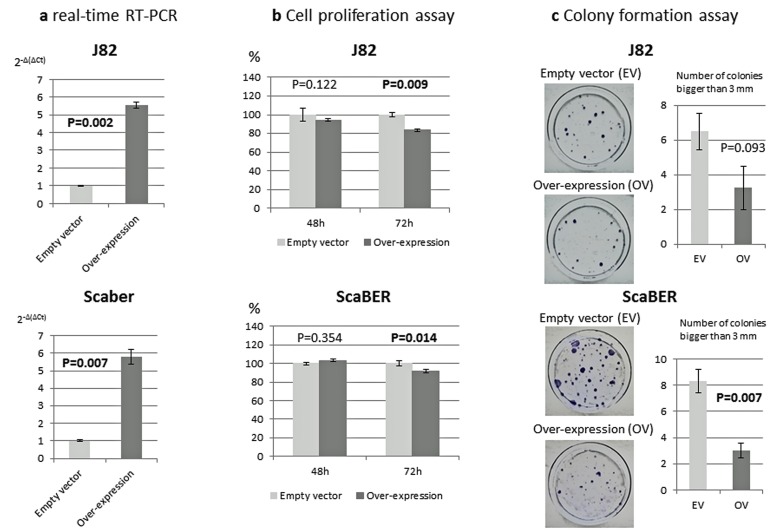
Phenotypic alterations of bladder cancer cells after ectopic expression of *VGF* **a.** *VGF* mRNA expression 24 hours after transfection to bladder cancer cell lines. Empty vector transfected cells showed no expression or little expression in comparison with *VGF* overexpressed cell; b. MTT assay: Significantly decreased numbers of J82 and SCaBER cells were observed 72 hours after forceful expression of *VGF* (P=0.009 and 0.014 respectively). Cell growth rate is expressed as absorbance at 570 to 650 nm; c. Colony formation assay: The effect of exogenous *VGF* expression in colony formation of J82 and SCaBER cell line. These cell lines were transfected with constructs encoding *VGF* or the empty vector. Cells were harvested 24 h after transfection, and equal cell numbers were seeded in 100 mm dishes and grown under selection in G418 for 21 days. The representative photograph of colonies of J82 and SCaBER cells 21 days after transfection with *VGF* construct or empty vector. As shown in the bar graph, number and size of the colonies are decreased after *VGF* overexpression (P=0.093 and 0.007 respectively, Student's t-test). Each assay was done in triplicate. Each bar graph represents the average ± standard error of three independent experiments.

To define the biologic consequences of *VGF* on cell proliferation and colony formation, we ectopically introduced *VGF* into two bladder cancer cell lines (J82 and SCaBER) and performed MTT and colony formation assays. *VGF* expression vector (pCMV6-AC–VGF–GFP, OriGene Technologies, Inc., Rockville, MD), as well as the empty vector (pCMV6-AC-GFP, OriGene Technologies, Inc.) were transfected into J82 and SCaBER bladder cancer cell lines that have fully methylated *VGF* promoter of our analyzed region and *VGF* silenced. Overexpression of *VGF* in comparison with empty vector was confirmed by real-time RT-PCR in J82 and SCaBER bladder cancer cell lines (Figure 3a). The results showed that ectopic expression of *VGF* decrease the viable cells as determined by MTT assay (P=0.009 and P=0.014 at 72 hours after transfection, Student's t-test) (Figure 3b). To observe the long term effect of *VGF* over-expression on cell growth, colony focus formation assay was performed and the result showed that the colony focus formed by *VGF*-transfected cells were less and smaller in size than those of empty vector transfected cells (P=0.093 and P=0.007 at 2 weeks after transfection, Student's t-test) (Figure 3c).

## DISCUSSION

Malignancies of bladder urothelium have distinct clinical and molecular characteristics. These cancers are often diagnosed at an early non-muscle invasive stage and are generally associated with favorable prognosis. However, a significant number of NMIBCs recur and a subset ultimately progress to invasive disease. The key similarities and differences between recurrent and progressive UCC and non-recurrent and non-progressive UCC have been compared from a molecular biology standpoint [25, 26]. However, the underlying mechanisms that determine the biological and molecular behaviors of UCC have not been fully elucidated. Thus, finding molecular target for early non-invasive detection, prognosis and therapeutic intervention for UCC is a promising avenue of research that may help to improve the survival of patients with this type of cancer.

Cancer related genes methylated in primary tumors at high frequency may serve as biomarkers for prognosis and early detection [27-30]. Cancer specific methylated genes may harbor tumor suppressive activity and methylation of some genes may alter key pathways that are related to initiation and progression of different cancer types [31-33]. The candidate gene approach we used in this study allowed us to identify UCC specific methylated genes with different frequencies (Supplemental Table S4) in cell lines. Although cell lines data may not reflect the methylation status in primary UCC due to genetic drift in culture conditions, these data allow us initial selection of genes for further studies in primary tumors. Furthermore, due to homogeneity, cell lines DNA provide clearer bisulfite sequencing readings that allow selection of CpG sites for developing high-throughput assays like MSP and QMSP for testing large number of primary tissue. However, although bisulfite sequencing is considered gold standard, it is less sensitive than pyrosequencing and QMSP.

We analyzed a total of 19 genes that may result in identifying interesting molecule for future studies related to UCC. For example, *HOXB5* gene encodes a sequence-specific transcription factor, and was reported to be methylated in ovarian carcinomas [34]; *MAL* gene methylation is also reported as a biomarker of cervical cancer [35], esophageal cancer [36] and colon cancer [37], it encodes a T-cell differentiation antigen, and has been shown to suppresses motility, invasion, and tumorigenicity and enhances apoptosis through the Fas pathway in esophageal cancer [38]; For *VGF* genes, in addition to reported cancer specific methylation in other solid tumors by our group [7-9, 17], we found complete methylation of the amplified region in both bladder cell lines and tissue samples. This initial observation prompted us to analyze primary UCC and urine by QMSP as a logical next step. To determine the UCC specific methylation, we initially analyzed a cohort of 19 tumor-normal paired tissues irrespective of stage and grade. As expected, we found higher level of methylation values in 16 of 19 UCCs in comparison with normal tissues. By our QMSP assay for *VGF*, some methylation values are also seen in matched normal samples, this could be due to field cancerization of the neighboring regions of the tumor lesions. As the purpose of this pilot study was to discover UCC specific methylation events, we did not analyze *VGF* QMSP data with clinicopathological parameters. Tumor depth related statistical analysis should be performed after testing a larger cohort of samples. Other 3 genes (CAMK4, RGS4 and ZMYM2) in Table 1 also need to be tested in primary tissues to determine their true UCC related sensitivity and specificity.

In order to determine whether *VGF* methylation is an early event for UCC initiation, we analyzed an independent cohort of 35 primary low grade papillary urothelial cell carcinomas (LGUCCs). Although the frequency of methylation was not as high as our randomly selected previous cohort of 19 cases, we found *VGF* methylation in a substantial number of LGUCC cases [12/35(34.^3^%)]. The discrepancy in methylation frequency in two different cohorts may be due to inclusion of higher stages and grades of tumors in initial cohort. Further studies using a larger cohort need to be performed to understand the true frequency of this gene in LGUCC and correlation of clinical outcome with *VGF* gene methylation. To understand any association of *VGF* methylation with clinicopathological parameters, well annotated clinical samples need to be tested that should include UCC samples of all grades and stages. However, in this study we tested a total of 54 UCC cases (19 with paired normal and 35 LGUCC) and our data showed reasonably high frequency of methylation of *VGF* and that warrant further methylation studies of this molecule in a larger cohort of samples.

DNA based molecular biomarker for non-invasive detection of UCC using urine sediments was first reported in 1991 [39]. As for methylation based biomarkers for UCC, promoter methylation of *DAPK, RAR*-β*, E-cadherin*, and *p16* were detected in 45.5%, 68.2%, 59.1%, and 13.6% of the 22 voided urine samples respectively [40]. In another study Dulaimi et al. [41] analyzed 45 UCCs and paired urine samples and reported consistency of promoter methylation of different genes between primary UCC and urine: 55.6% for *APC*, 40.0% for *RASSF1A* and 24.4% for *p14*. In the later study, even in the 21 low grade UCC cases, which are usually difficult to be detected by urine cytology (19.0%), methylation positive rates were 61.9% *for APC*, 52.4% for *RASSF1A* and 23.8% for *p14*. Quantitative methylation analysis has enabled to evaluate gene methylation status in a more sensitive and specific way [42, 43]. Friedrich et al [43] tested urine sediment DNAs from 37 UCC patients for a panel of 3 genes (*BCL2*, *TERT* and *DAPK*) and was able to detect methylation of at least one gene in 29/37 (78.4%) cases while no methylation was detected for any genes from the urine samples of control subjects. Similarly we also reported previously that UCC can be detected using urine sediment DNA with high sensitivity and specificity [27] and methylation patterns are consistent in primary UCC tissues and urine. Here we found high frequency of methylation of *VGF* in the urine of UCC cases [8/20(40%)] than controls [1/20(5%)]. We were not able to test methylation status of matched tumor-urine samples due to the lack of available samples. Thus, before the methylation screening test of a larger cohort of urine samples for *VGF* or a panel of methylated genes including *VGF*, a matched tumor-urine cohort need to be tested. Furthermore, urine samples from different stages and grades of UCC patients need to be tested to determine its utility as a screening/early detection marker.

In our study, promoter methylation of *VGF* inversely correlated with loss of gene expression in bladder cancer cell lines and primary UCC, and re-activation of *VGF* could be obtained by inhibiting DNA methylation with 5-Aza-dC. However, 5-Aza-dC may be non-specific and induce *VGF* expression may be due to alterations of related signaling pathways and demethylating other genes which regulate the expression of *VGF*. While additional studies are necessary to accurately understand the role of *VGF* gene silencing during the multistep process of tumorigenesis, our results argue that *VGF* may be one of the most frequent targets of unscheduled silencing induced by aberrant promoter methylation in the genesis of UCC. It is well established that DNA methylation of the CpG island in the promoter region is causally involved in gene silencing [7], therefore a tight correlation between *VGF* CpG island hypermethylation in cancer cell lines and loss of gene expression in these cells provides an explanation for the loss or inactivation of *VGF* previously reported in different pathological conditions [14]. Interestingly we found synergistic effect of 5-Aza-dC and TSA for the induction of *VGF* expression. Thus, studies on histone modifications on *VGF* may also be interesting.

Dysregulation of energy homeostasis is reported to be one of the major causes of carcinogenesis [46]. Many energy balance–related physiologic processes such as appetite, energy expenditure, body temperature control, energy metabolism regulated by hormones, cytokines, growth factors and inflammatory factors are altered in carcinogenesis. All the later physiologic processes are controlled by specific gene/genes that have influence on definitive pathways. For example, *RIP140* gene is known to have an important role in regulating lipid and glucose metabolism and RIP140 knockout mice are lean. Furthermore, this gene has influence on Wnt/APC/β-catenin signaling pathway [47]. In relation with cancer, *RIP140* mRNA expression was significantly decrease in human colon cancers, and forced expression of *RIP140* in cancer cells inhibits cell growth [47]. *VGF* gene also regulates energy homeostasis and metabolism[10]. VGF knockout mice also shows lean and hypermetabolic features [44]. *VGF* mRNA expression was significantly decrease in bladder cancers (Figure 1) and *VGF* over expression in bladder cancer cells inhibit cell growth. Based on the above information and our *VGF* data, we presume that it might be possible that restoration of homeostasis managing genes lead to some anti-cancerous effects on cancer cells even in *in vitro* setting. Further studies using *in vitro* and *in vivo* model systems are necessary to elucidate the correlation of *VGF* gene with major bladder carcinogenesis pathways including Ras-MARK signal transduction, p53 cell cycle regulation and retinoblastoma pathway [48].

In summary, this study enabled us to extract several UCC specific candidate methylated genes which is a logical progress to explore “UCC Methylome”. In an extended study using primary UCC tissues with paired normal, we successfully identified *VGF* as a UCC specific hypermethylated gene (P=0.009) and provided biologic evidence of growth inhibitory effect of *VGF* in bladder cancer cell lines. Furthermore, *VGF* is a potential marker for non-invasive detection of UCC using urine (P=0.020), although extended study using larger well annotated clinical cohort should be tested.

## MATERIALS AND METHODS

### Candidate gene selection

From the previously identified cancer specific methylated gene cohort [7], we first selected 19 candidate genes (*CAMK4*, *CKMT1b*, *FKBP4*, *GALE*, *HOXB5*, *HPN*, *KRT14*, *LPAR2*, *MAL*, *NBR1*, *NDP*, *NF1*, *PDLIM3*, *PHKA2*, *PVRL1*, *RGS4*, *SGK1*, *VGF* and *ZMYM2*) based on any one of the following criteria 1) Cancer specific methylation in any solid tumor we have tested previously; 2) No previous report of methylation in UCC; 3) Directly or indirectly related to key pathways that are known to be altered in UCC; and 4) known anti-proliferative activities.

### Cell Lines and Tissue Samples

Bladder cancer cell lines 5637, HT-1376, J82, SCaBER, SW780, T24, UM-UC3 and uro-epithelium cell line SV-HUC-1 were obtained from and propagated according to the recommendations of ATCC (Manassas, VA, USA). Mediums and antibiotics were purchased from Mediatech (Manassas, USA) and supplemented with fetal bovine serum (10%) (Hyclone, Logan, USA), 100 μg/ml streptomycin, and 100 I.U. /ml penicillin. Cells were grown at 37°C in a humidified atmosphere composed of 95% air and 5% CO_2_ in a monolayer culture. HT-1376, SCaBER and UM-UC3 were maintained in MEM; SW780 was grown in Leibovitz's L-15 medium; J82 were grown in DMEM; 5637 was maintained in RPMI 1640; T24 was grown in McCoy's 5A medium and SV-HUC-1 was grown in Ham's F-12K medium. Frozen human primary urothelial tumors and matched normal bladder tissue samples were kindly provided by Dr. D. Berman, Department of Pathology, The Johns Hopkins University School of Medicine. Urine samples were obtained from Department of Urology, The Johns Hopkins University School of Medicine. Approval for research on human subjects was obtained from The Johns Hopkins University Institutional Review Boards.

### DNA and RNA Extraction

Cell pellets were digested with 1 % SDS and 50 μg/ml proteinase K (Roche, Mannheim, Germany) at 48°C overnight. Isolation of genomic DNA from cell lines was performed with the phenol-chloroform extraction protocol followed by ethanol precipitation as previously described [20]. Bladder tissue samples were digested with 1 % SDS and 50 μg/ml proteinase K at 48°C for 2 days. DNA from tissue was extracted with phenol by using MaXtract High Density tubes (Qiagen, Valencia, CA, USA). For RNA extraction, adherent cells were detached by addition of RNA protect Cell Reagent (Qiagen), followed by the procedure of the RNeasy Plus Mini kit (Qiagen). RNA extraction from bladder tissue was performed with the QIAzol Lysis Reagent (Qiagen) following manufacturer protocol.

### Bisulfite-Modification of DNA, PCR Amplification and Sequencing Analysis

Sodium bisulfite-mediated conversion of unmethylated cytosines in DNA was performed with the EpiTect Bisulfite Kit (Qiagen). 2 g of cell line DNA and 0.5μg of bladder tissue DNA were processed. 2 μl bisulfite-converted genomic DNA was amplified by primers designed using the MethPrimer algorithm [21] (Supplemental Table S1). DNA was amplified for the 5' region including a portion of the CpG island within 1 kb of the transcriptional start site. Primer sequences were designed from the promoter regions without CpG dinucleotide. Platinum *Taq* DNA Polymerase (Invitrogen, Carlsbad, USA) were added and the reaction was carried out in a GeneAmp® PCR System 9700 (Life technologies, Carlsbad, USA). The annealing temperature of each gene was shown in Supplemental Table S1. PCR products were separated by electrophoresis on 1.5 % agarose gels stained with ethidium bromide and imaged in the Gel Doc XR with Quantity One Version 4.6.1. software (Bio-Rad, Hercules, USA). Upon visualization by electrophoresis, PCR products were extracted according to the QIAquick gel extraction protocol (Qiagen) and sequenced by the 3730xl DNA Analyzer (Life technologies) using the BigDye Terminator v3.1 cycle sequencing kit and forward or reverse primers. The data were analyzed using the Sequence Scanner v1.0 software (Life technologies). A methylation frequency of ≥ 50% of total CpG sites within the amplified region was considered “methylation-positive”.

### 5-Aza-2'-deoxycytidine (5-Aza-dC) Treatment of Cell Lines

Twenty four hours before treatment, cells were plated at low density (1-3×10^6^ cell concentration, dependent on growth characteristics of the respective cell line) in T25 or T75 cm^2^ Tissue Culture Flasks (BD Biosciences, Bedford, MA, USA). Stock solutions of 100 mM 5-Aza-dC (Sigma, St. Louis, USA) and 3 mM TSA (Sigma) were prepared with DMSO (Sigma). Immediately before addition to the cell culture medium, appropriate aliquots of the stock solutions were dissolved in PBS (pH 7.5). Cells were treated with 5 μM 5-Aza-dC (Sigma Chemical, Sigma, USA) for 3 to 5 days. Medium with 5-Aza-dC was changed daily. Additionally, combination treatment with 5-Aza-dC and TSA was performed by adding 5μM of 5-Aza-dC daily for 5 days and TSA (300 nmol/L; Sigma) was added to the medium for the final 24 hours. Cells were harvested 24 hours after the last day of treatment (5-Aza-dC only and 5-Aza-dC + TSA) for RNA extraction and the analysis of gene expression by Reverse Transcriptase-PCR (RT-PCR).

### Reverse transcription-PCR (RT-PCR) and Quantitative real time PCR (RT-QPCR)

Total RNA from cell lines and from bladder tissue were reverse-transcribed with SuperScript III First-strand Synthesis SuperMix (Life technologies) using random hexamers. 1 microliter of cDNA was subjected to PCR amplification. The primers were designed based on two different exons by means of Primer3 software [22]. Primer sequences and annealing temperatures were shown in Supplemental Table S2. Glyceraldehyde-3-phosphate dehydrogenase (*GAPDH*) was used as an internal control. The reactions were carried out as triplicates RT-QPCR. PCR products of cell lines were separated by electrophoresis on 1 to 1.5 % agarose gels stained with ethidium bromide and imaged in the Gel Doc XR (Bio-Rad). PCR products of bladder tissue samples were separated on PAGEr® Gold 10% TBE precast gels (Lonza, Rockland, ME, USA) with the addition of GelStar® Nucleic Acid Gel Stain (Lonza) to the loading buffer (Bio-Rad). For quantitative real-time PCR, the same pair of primers, SYBR® Green PCR Master Mix (Life technologies) and 7900HT real time PCR machine (Life technologies) were used. PCR conditions were 1 cycle: 95°C for 10 min; followed by 40 cycles: 95°C for 15 s and 60°C for 60 s. Expression of the gene of interest was quantified in triplicates relative to *GAPDH* using the 2-ΔΔCT method [23] for RT-QPCR.

### Methylation-specific PCR (MSP) and Quantitative MSP (QMSP)

Bisulfite-modified genomic DNA served as a template for MSP and QMSP. Primers were designed using the MethPrimer algorithm [21] and MSP primer algorithm [24]. *β-actin* served as a reference gene. QMSP primers and probes for *VGF* and *β-actin* were previously described [8] and also provided in Supplemental Table S3. Serial dilutions (90 − 0.009 ng) of CpG methyltransferase SssI-methylated (New England BioLabs, Ipswich, USA) human leukocyte genomic DNA from a healthy donor were used to construct a calibration curve for each plate. Amplification reactions were carried out in duplicate in a final volume of 20 μl containing 3 μl bisulfite-modified DNA, 600nmol/l forward and reverse primers, 200 nmol/l probe, 0.6 unit Platinum *Taq* DNA Polymerase (Invitrogen), dATP, dCTP, dGTP, and dTTP in a concentration of 200 μmol/l each, respectively, and 6.7 mmol/l MgCl_2_. Amplification reactions were carried out in 384-well plates in a 7900HT Fast Real-Time PCR System (Life technologies) and were analyzed by the Sequence Detector System software (SDS 2.3; Applied Biosystems). The relative level of methylated DNA for each gene in each sample was determined as a ratio of QMSP value of the amplified gene to *β-actin*, multiplied by 1000 for easier tabulation.

### Cell proliferation assay (MTT assay)

Bladder cancer cell lines J82 and SCaBER were plated on a 96-well plate at a density of 5 × 10^3^ to 1 × 10^4^ per well and incubated overnight at 37°C. The next day, cells were transfected with *VGF* expression vector (pCMV6-AC-VGF-GFP), empty vector (pCMV6-AC-GFP) (Origene, Rockville, MD) and FuGENE HD transfection reagent (Promega, Fitchburg, WI). Cellular viability was measured by the 3-(4, 5-dimethyl thiazol-2-yl)-2, 5-diphenyl tetrazolium bromide (MTT) proliferation assay kit (ATCC, Manassas, VA) according to the manufacturer's instructions after different time points. At the end of each time point, 10 μl of MTT labeling reagent (5 mg/ml MTT) was added to the culture medium, which was then incubated in the dark for a further 4 h at 37°C. This step was followed by cell lysis with the addition of 100 μl of a SDS-based detergent reagent. The plates were incubated for 2 h at 37°C to dissolve formazan crystals. Spectrophotometric readings (570 nm-650 nm) were obtained on a Spectra Max 250 96-well plate reader (Molecular devices, Sunnyvale, CA). Each assay was performed in triplicate, and each experiment was repeated at least three times. Data are represented as the extent of cellular survival expressed as a percentage of control (empty vector).

### Colony formation assay

Colony focus formation assays were performed as described previously [8]. Using *VGF* expression vector (pCMV6-AC-VGF-GFP), empty vector (pCMV6-AC-GFP) (Origene, Rockville, MD) and FuGENE HD transfection reagent (Promega); two bladder cancer cell lines (J82 and SCaBER) were transfected with each of the constructs. Twenty four hours after transfection, cells were divided into 3 dishes for each of the construct and G418 treatment was started in the following day. To confirm the expression of *VGF* in transfected cells, one additional dish of transfected cells were cultured for 24 hours, RNA was extracted and *VGF* expression was confirmed by reverse transcription-PCR (RT-PCR). After 2 weeks cells were washed twice with PBS, fixed with 25% acetic acid and 75% methanol at room temperature for 10 minutes and then stained with 0.1% crystal violet. Colonies were counted and the number of colonies per dish was averaged from three independent experiments (colonies >2 mm in diameter were considered as positive).

### Statistical Analysis

Continuous variables were analyzed by Student's t-test and categorical variables were analyzed by Fisher's exact test. All statistical analyses were performed using JMP 9 software (SAS institute, Cary, NC, USA). The level of statistical significance was set at P < 0.05 in two sided test.

## SUPPLEMENTARY FIGURE AND TABLES


