# Supplementary material for: Epigenetic inactivation of VGF associated with Urothelial Cell Carcinoma and its potential as a non-invasive biomarker using urine
Source: Oncotarget. 2014 May 7;5(10):3350–61. doi: 10.18632/oncotarget.1949 (PMC4102814; doi:10.18632/oncotarget.1949)
Supplement: Supplementary file 1 [file oncotarget-05-3350-s001.pdf]

Epigenetic inactivation of *VGF* associated with Urothelial Cell Carcinoma and its potential as a non-invasive biomarker using urine

Supplementary Materials

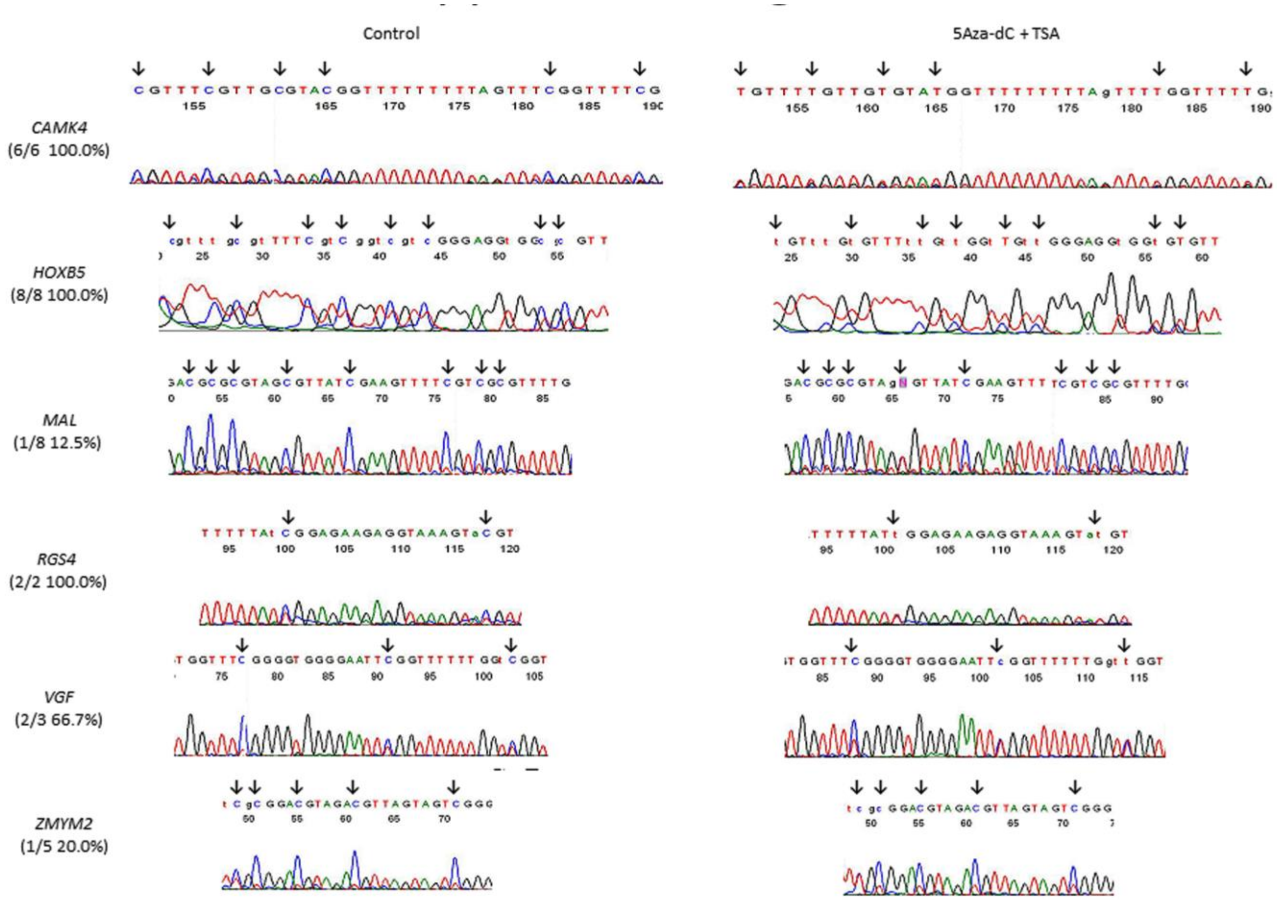

Supplemental Figure S1: Representative Electropherograms of direct sequencing of each gene's promoter region (*CAMK4*, *HOXB5*, *MAL*, *VGF* and *ZMYM2*) after bisulfite treatment of respective cancer cell line DNA; Left panel: Methylcytosine (arrows) present after bisulfite treatment in sense strand with vehicle treatment (controls); Right panel: cytosine converted to thymine (arrows) after treatment with 5-Aza-dC +TSA.

Supplemental Table S1: Primers used for Bisulfite Sequencing

RefSeq	Gene Symbol	F (5' - 3') (position at promoter)	R (5' - 3') (position at promoter)	Amplicon size (Nucleotide range)	Annealing temperature
**NM_001744**	*CAMK4*	TTTTGATAGAGGATGGTTGGTTAGT (□441 to □465)	AAACTTAAAAAAAACACAAAAACCC (□682 to □706)	266 bp (□441 to □706)	Step down 56-48°C
**NM_002147**	*HOXB5*	GTAGGGAGTTAGTAGGGAGGTAGT (□674 to □697)	AAATTAACTTTAACCTCTAAACAAAA (□757 to □782)	109 bp (□674 to □782)	Step down 56-48°C
**NM_002371**	*MAL*	TTTAATTGGGGTTAGATGTAGGTAG (□1391 to □1415)	AAAAACTTTAAAAAACCAAAAAAAA (□1645 to □1669)	279 bp (□1391 to □1669)	Step down 56-48°C
**NM_005613**	*RGS4*	TAGAGGGAGATAGAGGAGTTGGTATT (□904 to □929)	ACAAACCTACAAACCCTTTACACAT (□1125 to □1149)	246 bp (□904 to □1149)	Step down 56-48°C
**NM_003378**	*VGF*	AAGATTTATTTATTTGTTTTTGT (□330 to □352)	ACCAATAAAAACTCAATACT (□512 to □531)	202 bp (□330 to □531)	Step down 56-48°C
**NM_003453**	*ZMYM2*	GTTTTTTTTATTTTGGTTGATGGAG (□10 to □34)	TAAAAACTTAAAACCCCCTAAAAAC (□196 to □220)	211 bp (□10 to □220)	Step down 56-48°C

Supplemental Table S2: Primers used for RT-PCR

RefSeq	Gene Symbol	F (5' - 3') (position at coding sequence)	R (5' - 3') (position at coding sequence)	Amplicon size (Nucleotide range)	Annealing temperature
**NM_001744**	*CAMK4*	TCCATCGTGATCTCAAACCA (482 – 501)	CAGGTCCATAGGCACAACCT (642 – 661)	180 bp (482 – 661)	58°C
**NM_002147**	*HOXB5*	GCAGACTCCGCAAATATTCC (513 – 532)	TGTCCTTCTTCCACTTCATGC (737 – 757)	245 bp (513 – 757)	59°C
**NM_002371**	*MAL*	GGGTGATGTTCGTGTCTGTG (252 – 272)	ACACCATCTGGGTTTTCAGC (540 – 559)	308 bp (252 – 559)	60°C
**NM_005613**	*RGS4*	AGTCCCAAGGCCAAAAAGAT (322 – 341)	GACGGGTTGACCAAATCAAG (523 – 542)	221 bp (322 – 542)	59°C
**NM_003378**	*VGF*	GACCCTCCTCTCCACCTCTC (160 – 179)	ACCGGCTCTTTATGCTCAGA (325 – 345)	186 bp (160 – 345)	62°C
**NM_003453**	*ZMYM2*	GGGGAAAACAGAGACAACCA (2883 – 2902)	GGTCTGGGCTGTTCCTCATA (3097 – 3116)	234 bp (2883 – 3116)	58°C

Supplemental Table S3: Primers and Probes used for QMSP

RefSeq	Gene Symbol	F (5' - 3') (position at promoter)	R (5' - 3') (position at promoter)	Probe (6FAM 5' - 3'TAMRA)	Amplicon size (Nucleotide range)	Annealing temperature
**NM_003378**	*VGF*	GGATAGCGTTCGTAGGCG (–502 to –483)	AAAAACCGAATTCCCCACCCCG (–430 to –418)	GCGCCCAAAAACGACGTAAACCTAAATAC (–465 to –447)	85 bp (–502 to –418)	60.0°C

Supplemental Table S4: Candidate genes with high frequency of methylation in bladder cancer cell lines based on bisulfite sequencing

RefSeq	Gene Symbol	Gene Name	Locus	Positive Frequency in cell lines	
NM_001744	*CAMK4*	calcium/calmodulin-dependent protein kinase IV	5q21.3	2/6	33.3 %
NM_020990	*CKMT1b*	creatine kinase, mitochondrial 1B	15q15	3/7	42.9 %
NM_002014	*FKBP4*	FK506 binding protein 4	12p13.33	5/7	71.4 %
NM_000403	*GALE*	UDP-galactose-4-epimerase	1p36-p35	7/7	100.0 %
NM_002147	*HOXB5*	homeobox B5	17q21.3	3/7	42.9 %
NM_002151	*HPN*	hepsin	19q11-q13.2	5/7	71.4 %
NM_000526	*KRT14*	keratin 14	17q12-q21	6/7	85.7 %
NM_004720	*LPAR2*	lysophosphatidic acid receptor 2	19p12	2/7	28.6 %
NM_002371	*MAL*	T-cell differentiation protein	2cen-q13	7/7	100.0 %
NM_031858	*NBR1*	neighbor of BRCA1 gene	17q21.31	1/6	16.7 %
NM_000266	*NDP*	Norrie disease (pseudoglioma)	Xp11.4	3/7	42.9 %
NM_000267	*NF1*	neurofibromin 1	17q11.2	7/7	100.0 %
NM_014476	*PDLIM3*	PDZ and LIM domain 3	4q35	2/6	33.3 %
NM_000292	*PHKA2*	phosphorylase kinase, α2	Xp22.2-p22.1	1/6	16.7 %
NM_002855	*PVRL1*	poliovirus receptor-related 1	11q23.3	1/6	16.7 %
**NM_005613**	*RGS4*	regulator of G protein signaling 4, isoform 2	1q23.3	2/6	33.3 %
**NM_005627**	*SGK1*	serum/glucocorticoid regulated kinase 1	6q23	1/6	16.7 %
**NM_003378**	*VGF*	VGF nerve growth factor inducible	7q22	4/7	57.1 %
**NM_003453**	*ZMYM2*	zinc finger, MYM-type 2	13q11-q12	4/7	57.1 %

Supplemental Table S5: Promoter DNA methylation status of 6 candidate genes in 7 bladder cancer cell lines based on bisulfite sequencing

RefSeq	Gene Symbol	5637	HT-1376	J82	SCaBER	SW780	T24	UM-UC3
**NM_001744**	*CAMK4*	U	M	U	M/U	n.a.	U	U
**NM_002147**	*HOXB5*	M	U	M	U	U	U	M
**NM_002371**	*MAL*	M	M/U	M/U	M	M	M	M
**NM_005613**	*RGS4*	M	U	M	U	n.a	U	U
**NM_003378**	*VGF*	M	U	M	M	U	M	U
**NM_003453**	*ZMYM2*	U	M	U	U	M	M	M

M: methylated, U: unmethylated, M/U: both methylated and unmethylated, n.a.: not assessed

Supplemental Table S 6: Clinicopathological characteristics of tested urine samples of bladder cancer patients

Sample number	Cytology	Cystoscopy	Tumor depth	Tumor grade	VGF QMSP
307	n.a.	n.a.	n.a.	n.a.	Positive
308	n.a.	n.a.	n.a.	n.a.	Positive
309	n.a.	n.a.	n.a.	n.a.	Positive
314	Positive	Positive	NMIBC	Low grade	Negative
316	Positive	Positive	NMIBC	Low grade	Negative
317	Positive	Positive	MIBC	Low grade	Positive
320	Positive	Negative	NMIBC	Low grade	Negative
322	Negative	Positive	MIBC	Low grade	Negative
323	Positive	n.a.	MIBC	Low grade	Positive
324	Negative	Negative	MIBC	Low grade	Negative
325	n.a.	n.a.	n.a.	n.a.	Positive
326	Negative	Negative	n.a.	n.a.	Negative
327	n.a.	n.a.	n.a.	n.a.	Negative
328	n.a.	n.a.	n.a.	n.a.	Negative
329	n.a.	Positive	NMIBC	High grade	Positive

330	Positive	Positive	MIBC	Low grade	Negative
**331**	n.a.	n.a.	n.a.	n.a.	Negative
**332**	Positive	n.a.	NMIBC	Low grade	Positive
**333**	Positive	Negative	MIBC	Low grade	Negative
**335**	n.a.	Positive	MIBC	Low grade	Negative

MIBC: muscle invasive bladder cancer, NMIBC: non muscle invasive bladder cancer, n.a.: no information available
